# An Unusual Superior Root of the Ansa Cervicalis

**DOI:** 10.7759/cureus.4558

**Published:** 2019-04-27

**Authors:** Shogo Kikuta, Joe Iwanaga, Jingo Kusukawa, R. Shane Tubbs

**Affiliations:** 1 Seattle Science Foundation, Seattle, USA; 2 Medical Education and Simulation, Seattle Science Foundation, Seattle, USA; 3 Dental and Oral Medical Center, Kurume University School of Medicine, Kurume, JPN; 4 Neurosurgery, Seattle Science Foundation, Seattle, USA

**Keywords:** ansa cervicalis, anatomy, neck surgery, variation, vagus nerve

## Abstract

The ansa cervicalis is located around the carotid sheath and forms a neural loop, which consists of superior and inferior roots. It innervates the infrahyoid muscles. Anatomical variations of the superior root of the ansa cervicalis are uncommon. Herein, we present an extremely rare case of the superior root of the ansa cervicalis arising both from the hypoglossal and vagus nerves.

## Introduction

The ansa cervicalis is located deep into the sternocleidomastoid muscle and innervates the infrahyoid muscles. It is formed by two roots, superior and inferior. The fibers from the ventral rami of the first and second cervical spinal nerves (C1-C2) hitchhike along the hypoglossal nerve (HN) for a distance of 3-4 cm to become the superior root of the ansa cervicalis [1-3]. The superior root consistently leaves the HN and descends along the anterior wall of the carotid sheath. The inferior root arises from the ventral rami of the second and third cervical spinal nerves (C2-C3). The superior and inferior roots join to form a neural loop anterior to the internal jugular vein (IJV), in most cases [4]. The inferior root of the ansa cervicalis has a multitude of anatomical variants [3,5-10]. To our knowledge, only a few variants of the superior root have been reported. Herein, we report an unusual contribution of the vagus nerve to the superior root of the ansa cervicalis.

## Case presentation

**Figure 1:**
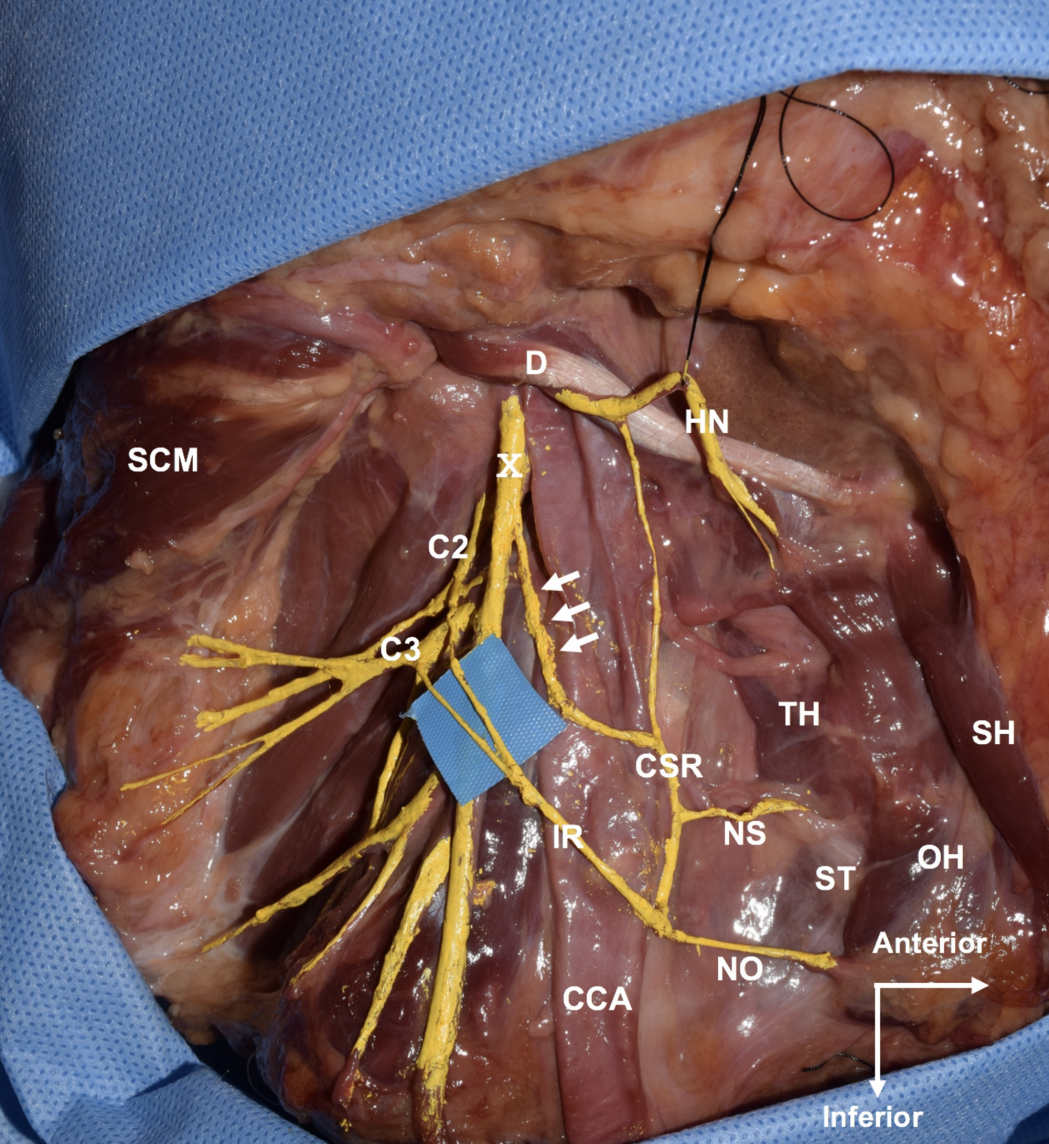
Lateral view of the right neck. Note the branch from the vagus nerve (arrows) joins the branch from the hypoglossal nerve to form the common superior root CCA, common carotid artery; CSR, common superior root; D, posterior belly of digastric; HN, hypoglossal nerve; IR, inferior root; NO, nerve to superior belly of omohyoid; NS, nerve to sternothyroid; OH, superior belly of omohyoid; SCM, sternocleidomastoid muscle; SH, sternohyoid; ST, sternothyroid; TH, thyrohyoid; X, vagus nerve.

During the routine cadaveric dissection of a fresh-frozen cadaveric neck we identified a variant right superior root of the ansa cervicalis. The cadaver was a Caucasian female whose age at death was 77 years. The superior root of the ansa cervicalis was formed by two distinct branches: one descending from the HN and one arising from the vagus nerve at the level of C3 vertebra. Both branches descended along with the anterior wall of the carotid sheath and united to form the common superior root at the level of the common carotid artery bifurcation. The inferior root was formed by the ventral rami of the second and third cervical spinal nerves and descended to form a loop together with the superior root; as a result, the ansa cervicalis consisted of two loops. Nerve supply to the sternothyroid and omohyoid from the ansa cervicalis was observed. Both roots were located deep to the IJV (Figure 1). No other anatomical anomalies or previous surgical scars were found in the dissection area.

## Discussion

The ansa cervicalis was historically known as the ansa hypoglossi as it arises from the HN [11]. Also, historically, the superior root of the ansa cervicalis has been called the "descendens hypoglossi" [12]. The superior root consists of fibers arising from the C1-C2 and does not include fibers from the HN [2]. Roots of the ansa cervicalis travel along multiple paths and have various origins. Jelev [9] suggested a classification of the ansa cervicalis (type I to V) with possible communication from three sources: the C1-C2 fibers via the HN (hypoglossal nerve component), the C1-C2 fibers within the vagus nerve (vagal component), and separate branches from the C2-C3 ventral rami (cervical component). The author described that in a typical ansa cervicalis, the superior root has a hypoglossal component and the inferior root has a cervical component, concluding its variation is less than 1% based on a review of the literature.

Rath and Anand reported the absence of the ansa cervicalis with the innervation of the infrahyoid muscles being replaced by a branch of the vagus nerve in one case of 400 cadavers [6]. The variant formation was termed a “vagocervical plexus” or “vagocervical complex” [6-7]. In the present case, the common superior root was formed by the descendens hypoglossi and the branch from the vagus nerve, which joined the inferior root formed the ansa. To our knowledge, only two cases of a vagal contribution to the superior root have been reported [10,13]. Nayak et al. [10] proposed that this formation be called a “descendens vagohypoglossi.” In the present case, the descending branch from the vagus nerve might have “hitchhiked” via the C1-C2. Although the diameter was not measured, the contribution from the vagus nerve to the common superior root seems to be larger than that of the hypoglossal nerve. The vagus nerve might have communicating branches with C1 and/or C2 proximally, which might compensate for the smaller contribution from the hypoglossal nerve. Previous authors have mentioned that the branch from the vagocervical plexus compensates for the nerve supply to the infrahyoid in cases where the ansa cervicalis is absent [7]. This variant of the ansa cervicalis is extremely rare, but knowledge of its existence is necessary during the operations in this area as for example, unintended traction on the vagus nerve could occur with dissection of the ansa cervicalis.

## Conclusions

Only a few previous reports describe variations of the superior root of the ansa cervicalis. We have reported a rare formation of the superior root contributed to by the vagus nerve.
